# Design of a New Seismoelectric Logging Instrument

**DOI:** 10.3390/s21248489

**Published:** 2021-12-20

**Authors:** Liangchen Zhang, Xiaodong Ju, Junqiang Lu, Baiyong Men, Weiliang He

**Affiliations:** 1 State Key Laboratory of Petroleum Resources and Prospecting, Key Laboratory of Earth Prospecting and Information Technology, China University of Petroleum, Beijing 102249, China; juxdong@cup.edu.cn (X.J.); lujq@cup.edu.cn (J.L.); bymen@cup.edu.cn (B.M.); 2 China Electronic Technology Group Corporation Twentieth Research Institute, Xi’an 710068, China; 18201426471@126.com

**Keywords:** seismoelectric logging instrument, phased linear array, electronic system, sonde complex, data processing

## Abstract

To increase the accuracy of reservoir evaluation, a new type of seismoelectric logging instrument was designed. The designed tool comprises the invented sonde-structured array complex. The tool includes several modules, including a signal excitation module, data acquisition module, phased array transmitting module, impedance matching module and a main system control circuit, which are interconnected through high-speed tool bus to form a distributed architecture. UC/OS-II was used for the real-time system control. After constructing the experimental measurement system prototype of the seismoelectric logging detector, its performance was verified in the laboratory. The obtained results showed that the consistency between the multi-channel received waveform amplitude and benchmark spectrum was more than 97%. The binary phased linear array transmitting module of the instrument can realize 0° to 20° deflection and directional radiation. In the end, a field test was conducted to verify the tool’s performance in downhole conditions. The results of this test proved the effectiveness of the developed seismoelectric logging tool.

## 1. Introduction

Seismoelectric logging is an emerging geophysical logging method. The seismoelectric coupling effects between an elastic wave and an electromagnetic field have potential applications in oil-gas exploration [1]. At present, the theory and application of seismoelectric logging is an important research and development subject. Frenkel carried out a theoretical study on the seismoelectric effect and proposed the wave transmission equations for porous media [2]. Based on Biot’s acoustic theory of porous media, the Pride theory of the seismoelectric coupling of porous media was obtained [3,4]. Hu et al. simulated the time domain of acoustic pressure waveforms and axial electric fields along the well axis by solving Pride equations [5]. Guan et al. used a finite difference algorithm to simulate the seismoelectric hole waveform in fluid-filled borehole [6]. Singer et al. used the finite element method to simulate the acoustic logging wave field [7]. Grobbe et al. simulated the seismoelectric logging wave field in a horizontally layered formation [8]. Some models were presented to describe the propagation of waves in a porous medium saturated by compressible fluid [9,10]. MIT scholars carried out relevant experiments on electrokinetic logging [11,12]. They built a small well model in the laboratory, and observed two types of waves using a sound excitation source. Some studies measured the flow potential and seepage current of core samples through experiments, and obtained the electro-dynamic coupling coefficient [13]. Mikhailov measured the accompanying electric field generated by low-frequency Stoneley acoustic signals in the well [14]. The seismoelectric laboratory experiment was designed to quantify the seismoelectric transfer function [15]. Considering the sensitivity of an induced electric field to fluid salinity, Wang et al. simulated the time-domain waveforms of electric fields for different pore fluid salinity levels [16]. Peng et al. studied the influence of rock size and shape on the interface seismoelectric response by measuring signals in a wedge rock model [17]. The influence of rock permeability and porosity on seismoelectric conversion was also discussed [18]. An experimental setup was designed to evaluate rock permeability by measuring the streaming current [19]. Zhang et al. studied the waveform and velocity characteristics of interface seismoelectric signal [20].

While physics experiments and numerical simulations are the main research directions in seismoelectric logging, the development of downhole electrokinetic logging tools is typically left out of scope. At the 43rd SPWLA logging annual meeting, the OYO Company of Japan and the Ground-Flow Company of the UK jointly proposed the EKL logging instrument based on the principle of the seismoelectric effect [21]. The instrument was tested in water wells, and the water resistivity and permeability of formation were obtained. The acoustic logging laboratory of the China University of Petroleum developed a seismoelectric logging tool which was compatible with the Eilog imaging system of the China Petroleum Logging Company [22]. The whole detector was connected to the Eilog system during the field testing, and ideal results were obtained [23]. The China University of Petroleum developed a new generation of seismoelectric logging detectors, which are currently undergoing laboratory and field tests [24,25,26]. Guan et al. proposed a method to invert permeability from seismoelectric logs in fluid-saturated porous formations, which has a guiding effect on actual data processing [27,28].

This paper describes the design of the newly proposed seismoelectric logging detector and the results of the corresponding field and experimental studies, which create a framework for subsequent tool and data processing improvements.

## 2. Structural Design of Seismoelectric Logging Tool

### 2.1. Overall Design of the Tool

**Figure 1:**
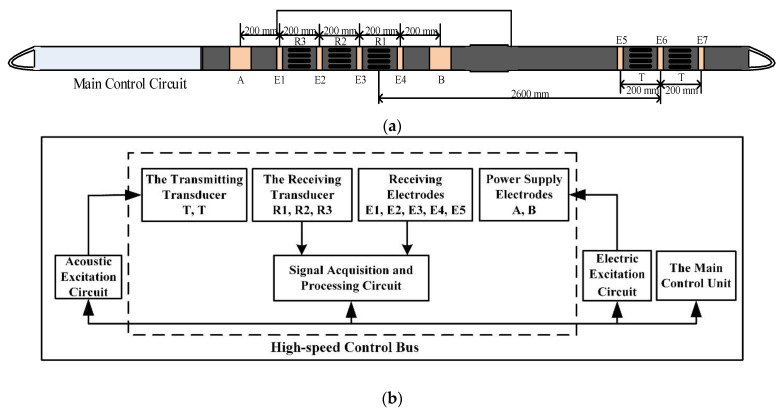
(**a**) Structure diagram of the seismoelectric logging detector. (**b**) The internal electronic system of the instrument.

The new generation of seismoelectric logging tools developed by the China University of Petroleum includes an acoustic signal excitation module, an acoustic signal receiving module, and electrical signal receiving and excitation modules. It has the functionality of a traditional downhole acoustic detector and an electrical detector. The structure diagram of the instrument is shown in Figure 1a, and the electronic system diagram is shown in Figure 1b.

Each functional module of the electronic system is interconnected through the internal high-speed bus. The seismoelectric logging tool includes two acoustic excitation sources, three acoustic receiving stations, two electrical excitation electrodes and seven electrical receiving electrodes. Both the transmitting transducer and the receiving transducer are located in the short section of the composite detector, which improves the quality of signal acquisition and shortens the bottom length of the instrument. The structure of the transmitting transducer is a binary phased linear array, and each array element is composed of three parallel high-power monopole transmitting transducers. The instrument has three high-sensitivity monopole receiving transducers, the center distance is 200 mm, and the center distance between the transmitting transducer and the nearest receiving transducer is 2600 mm. There are four array receiving electrodes (*E1*, *E2*, *E3* and *E4*) around the receiving transducer. The size of the electrode system can be expressed as follows:

(1) $$\frac{0.1}{A}0.2\frac{0.02}{E4}0.2\frac{0.02}{E3}0.2\frac{0.02}{E2}0.2\frac{0.02}{E1}0.2\frac{0.1}{B}$$

The width of the measuring electrode (*E1*, *E2*, *E3*, *E4*) is 0.02 m and the center distance of each electrode is 0.2 m. *A* and *B* are power supply electrodes, and the electrode width is 0.1 m.

The seismoelectric logging tool extends the bandwidth of the electrical measurement channel to 20 kHz, which enables induced polarization and spontaneous potential measurement. The main electrode has both a potential measurement mode and a differential measurement mode to improve the signal-to-noise ratio of the acquisition signal. The instrument has three receiving electrodes (*E5*, *E6*, *E7*) around the transmitting transducer. The center distance between the electrodes is 200 mm. *E6* registers potential, and the combination of *E5* and *E7* receives the signal in the differential mode. In order to insulate the electrodes from each other, the short section of the composite detector has a glass steel shell, while the main control electronic circuit has a titanium steel alloy shell.

**Figure 2:**
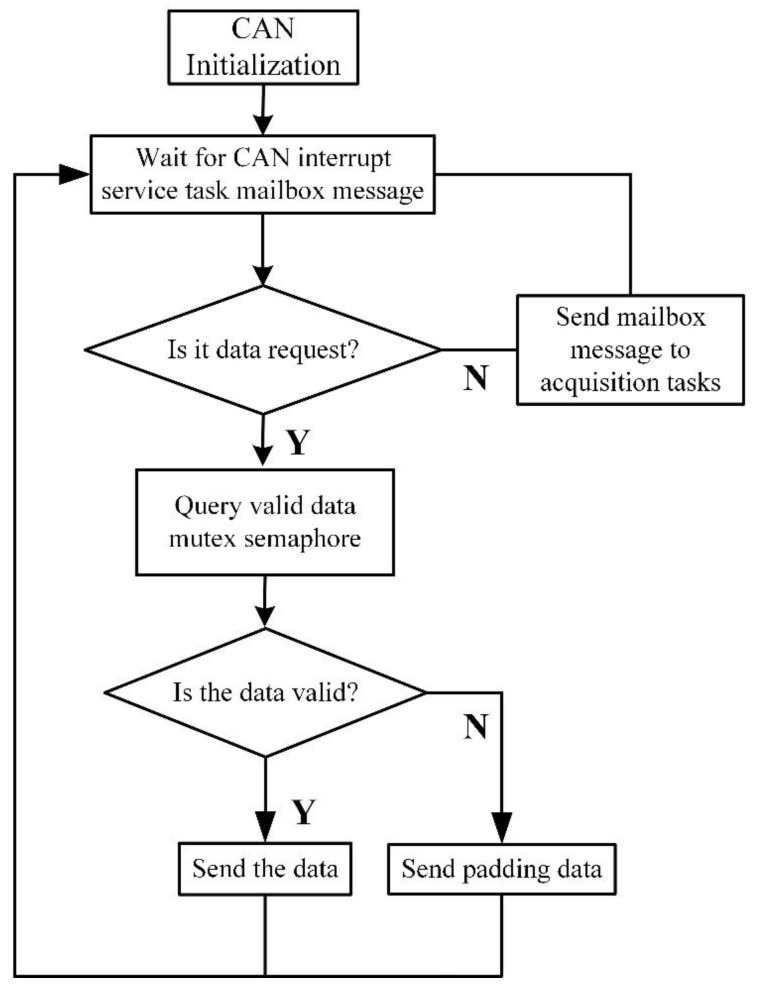
The process of the CAN communication workflow.

The instrument runs the main control software in a DSP (digital signal processor), and uC/OS-II is used to achieve real-time system control. Such a design has the advantages of high execution efficiency, less resource consumption and strong real-time performance, which are convenient for real-time control applications with limited underground resources. There are two main tasks running on the uC/OS-II: the CAN (controller area network) communication task and the work cycle control. The CAN communication task mainly realizes the initialization of the CAN controller and the communication with telemetry sub. The process of the CAN communication workflow is shown in Figure 2. The work cycle control task is mainly used to control the high-speed interconnection bus and the instrument work cycle.

### 2.2. Design of the Excitation Circuit

**Figure 3:**
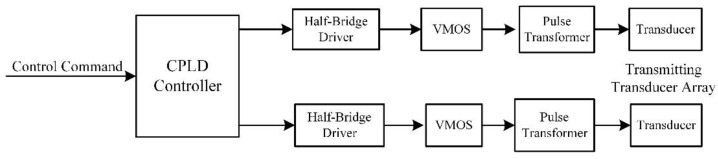
Schematic diagram of the acoustic excitation module circuit.

The excitation circuit includes acoustic and electric excitation circuits. The acoustic excitation circuit module has two independent high-power pulse channels which are used to improve the excitation for two groups of high-power acoustic transducers. The excitation mode of the phased linear array can be formed through reasonable inter group delay, which is beneficial to improve the signal-to-noise ratio of the converted wave. A CPLD (complex programmable logic device) is used as the interface and the logic controller of the transmitting circuit board is used to control the acoustic excitation time, pulse width and phase control delay. The schematic diagram of the excitation module circuit is shown in Figure 3.

**Figure 4:**
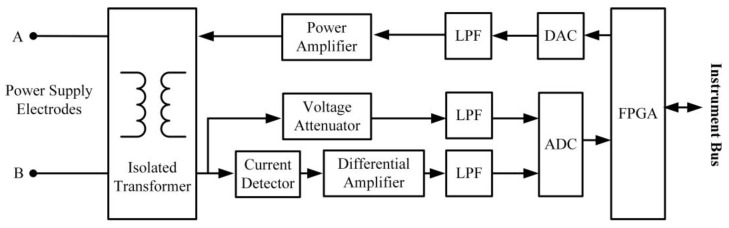
Schematic diagram of the electric excitation module circuit.

The electric excitation module is a high-precision arbitrary analog waveform generator. The circuit diagram is shown in Figure 4. The control chip of the module performs the data interface with the main control module through the dedicated serial bus. Under the selection of the main control command parameters, it generates the required analog waveform signal by scanning and outputting the internal waveform data table to the DAC. We amplify the power of the signal after low-pass filtering, then output it to the power supply electrodes through the isolation transformer to achieve electrical excitation. The electrical excitation signal has the same main frequency as the working frequency of the acoustic receiving transducer. The intensity of the excitation signal is monitored in real time. Before the formal electrical excitation, several trial excitations can be carried out to adjust the power to adapt to different formations and thus achieve the best undistorted electrical excitation. The actual excitation parameters are recorded and used as the calculation basis in the computation of the apparent resistivity and in the electro-acoustic conversion wave processing.

### 2.3. Signal Acquisition and Processing Module

Theoretical calculations and experimental results show that the converted wave signal is quite weak. To solve this problem, a multi-channel parallel analog signal processing and data acquisition circuit with high signal-to-noise ratio and large dynamic range was designed for seismoelectric logging instruments.

**Figure 5:**
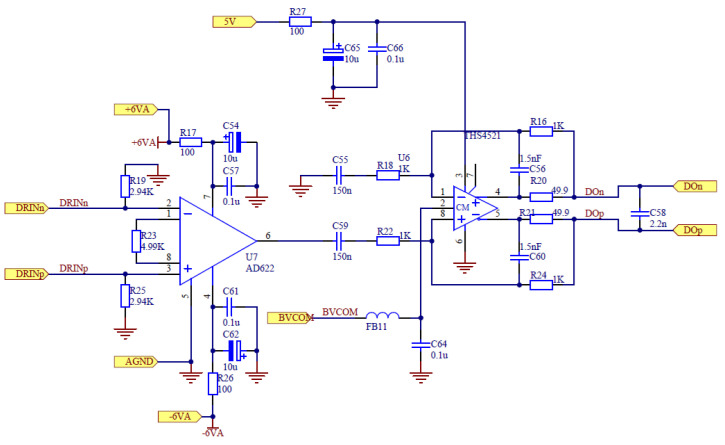
The preamplifier circuit of the tool.

The main acquisition mode of the instrument has 6 channels, including three acoustic wave receivers and three electrical differential receivers. The low noise instrument amplifier achieves a fixed gain of 40 dB to obtain a high signal-to-noise ratio when the signal is weak. The auxiliary acquisition mode uses 4-channel potential measurement, and the gain of pre-amplifier is 10 dB. We used an instrumentation amplifier as a low gain preamplifier. In the case of high gain, the noise was small. The AD622 chip was selected as the preamplifier, and the instrument’s amplifier circuit is shown in Figure 5. This chip has low input and output voltage noise density and good noise suppression performance. The common mode suppression ratio and power supply noise suppression ratio are high. At the same time, the gain setting of the chip is simple and a single resistor can be used to control the gain. The corresponding gain formula is given by Equation (2). According to this equation, the gain reaches the value of 11 when *R_G_* is set to 4.99 kΩ.

(2) $$G=1+50.5k/R_{G}$$

The main and auxiliary acquisition mode signals are selected by the analog multiplexer and introduced into the amplification filter channel. The six parallel amplification and filtering channels compose the general analog signal conditioning channel of the instrument. Each channel is composed of a two-stage programmable gain amplifier (PGA) and a band-pass filter (BPF). The combination of PGA and BPF can achieve continuous 6 dB step adjustments in the range of 0 to 90 dB to adapt to the large dynamic range of the acoustoelectric converted wave signal. Through the BPF, the signal frequency band is limited to a reasonable range to ensure the signal-to-noise ratio. The passband range of the pass-band filter is 5 to 22 kHz. The filter effectively improves the signal-to-noise ratio and enables the system to have an anti-aliasing dynamic range of 140 dB. We used an analog-to-digital converter to achieve six-channel parallel synchronous data acquisition with a resolution of 16 bits and a shortest sampling interval of 2 μs. As the main control logic device of the signal amplification and acquisition module, FPGA performs the mode selection, channel gain control, data cache and other functions. The working parameters of the signal amplification and acquisition module are processed by the main control module and set through the instrument control bus. A typical acoustic excitation mode data frame includes 10 channels of data with an 8-microsecond sampling interval and a sampling depth of 512 words per channel, which can effectively obtain the acoustic and electrical full wave signals of seismoelectric logging.

### 2.4. Impedance Matching Design of Transducer

**Figure 6:**
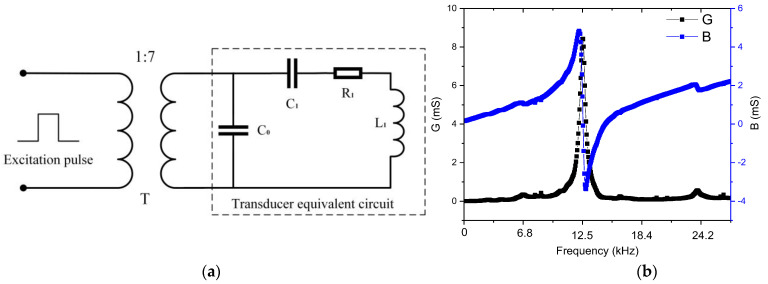
(**a**) Equivalent circuit of transformer excitation transducer. (**b**) Test results of transducer impedance.

The signal collected by the seismoelectric logging instrument is very weak. During the detection process, a downhole instrument needs to transmit a sufficiently powered acoustic signal into the formation. It is necessary to use a high-power pulse signal to excite the transmitter transducer. At the same time, impedance matching should be achieved between the signal source and the transducer to effectively improve the excitation efficiency of the signal source to the transducer and to improve the signal-to-noise ratio. As shown in Figure 6a, the pulse signal directly excites the transducer after boosting through a pulse transformer, and the transducer can be equivalent to an impedance network composed of *C*_0_, *C*_1_, *R*_1_ and *L*_1_, where *C*_0_ is the static capacitance of the transducer, *C*_1_ is the dynamic capacitance, *R*_1_ is the dynamic resistance and *L*_1_ is the dynamic inductance. At present, impedance matching technologies have focused on single frequencies, and the impedance characteristics can be expressed by Equation (3) [29].

(3) $$Y=\frac{jw^{2}C_{1}R_{1}C_{0}-wC_{0}\left(w^{2}L_{1}C_{1}-1\right)+wC_{1}\;}{wR_{1}C_{1}+j\left(w^{2}L_{1}C_{1}-1\right)}$$

**Figure 7:**
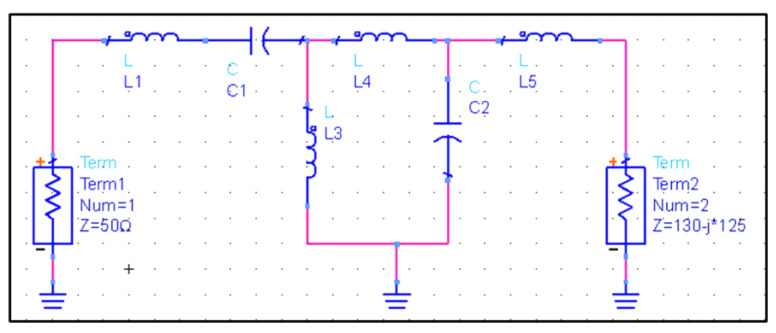
The structure of impedance matching network designed by ADS software.

In this paper, we used the Smith chart method for impedance matching. A Smith chart is an impedance matching method based on the theory of power transmission. By adding inductance and capacitance to the impedance matching network, the load and excitation source achieve conjugate matching. The performance of the impedance matching network is measured by the reflection coefficient S11. The smaller the reflection coefficient, the higher the power transmission efficiency of the system. The Smith chart module in ADS software was used to design the structure of impedance matching network. It can be seen in Figure 6b that the resonant frequency is 13.5 kHz. Using ADS software, the impedance data, 130–125 j of 13.5 kHz, was taken as the target load, and the impedance of the signal source was 50 Ω. The structure of the designed impedance matching network is shown in Figure 7.

### 2.5. Design of Phased Array Radiator

The amplitude of the received signal in seismoelectric logging is small, so it is necessary to improve the intensity of transmitted signal using an appropriate technology. According to the literature [30,31,32,33,34,35], a phased array acoustic logging radiator can improve the radiated sound field, the signal-to-noise ratio of received signal and the detection depth. Therefore, a phased array radiator is also included into this instrument. At present, there are many array element spacing design methods, among which the dynamic programming method can realize a phased array design with a small number of array elements.

**Figure 8:**
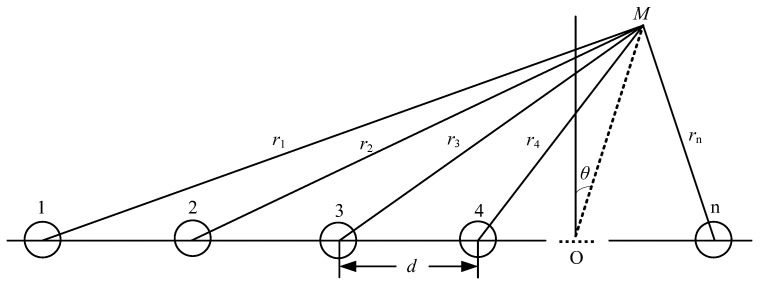
Diagram of a linear array sound source.

In this paper, the numerical simulation method was used to optimize the array element spacing of the phased array radiator instrument. Optimal array element spacing and number of array elements were obtained to minimize the side lobe of phased linear array radiator, which improved the optimization parameters for the instrument design. A linear array sound source is composed of multiple point sound sources and spacing in a straight line (see Figure 8). The phased array radiator can be formed by delaying its working time.

Equation (4) presents the sound pressure formula for *n* point source synthesis:

(4) $$p=\frac{A}{r_{1}}e^{j\left(wt-kr_{1}\right)}+\frac{A}{r_{2}}e^{j\left(wt-kr_{2}\right)}\ldots \frac{A}{r_{n}}e^{j\left(wt-kr_{n}\right)}$$

After derivation and simplification, the sound pressure is expressed as:

(5) $$p=\frac{A}{r}e^{j\left(wt-kr\right)}\frac{sin\left(\frac{nkd}{2}sin\theta \right)}{sin\left(\frac{kd}{2}sin\theta \right)}$$

From here, the sound source directivity function can be defined as:

(6) $$D\left(\theta \right)=\left|\frac{sin\left(\frac{nkd}{2}sin\theta \right)}{nsin\left(\frac{kd}{2}sin\theta \right)}\right|$$

where *n* is the number of array elements, *d* is the element spacing, and *k* is the wave number. According to the general expression of the directivity function, directivity diagrams with the different number of elements (*n* = 3, 4, 5, 6) were calculated, respectively. It can be found that the sidelobe level can be reduced with fewer array elements.

**Figure 9:**
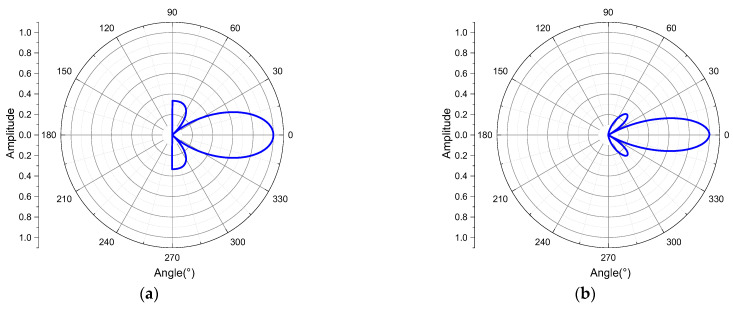

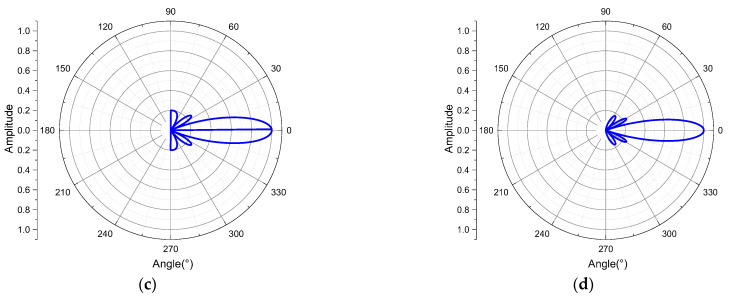
Directivity graphs with different numbers of array elements. (**a**) *n* = 3; (**b**) *n* = 4; (**c**) *n* = 5; (**d**) *n* = 6.

According to the results shown in Figure 9, when the number of array elements is 4, the sidelobe is smaller and the directivity is better. However, considering the difficulty of the implementation of the instrument circuit, we designed a transmitting transducer structure containing a two-element linear phased array, in which each array element consists of three high-power monopole transmitting transducers in parallel. 

## 3. Evaluation of the Performance of the Transducer of the Seismoeletric Logging Tool

After the electronic system of the seismoelectric logging detector was debugged and assembled in the laboratory, in order to study the intensity and directivity of the acoustic field excited by the acoustic emission transducer of the detector and the practical application effect of the phased linear array technology, we carried out experiments on the radiation sound field of the detector in a pool. This was a comprehensive test of the electronic system and working performance of the detector, as well as a laboratory verification of the entire design scheme.

### 3.1. Experimental System of the Transmitting Transducer

**Figure 10:**
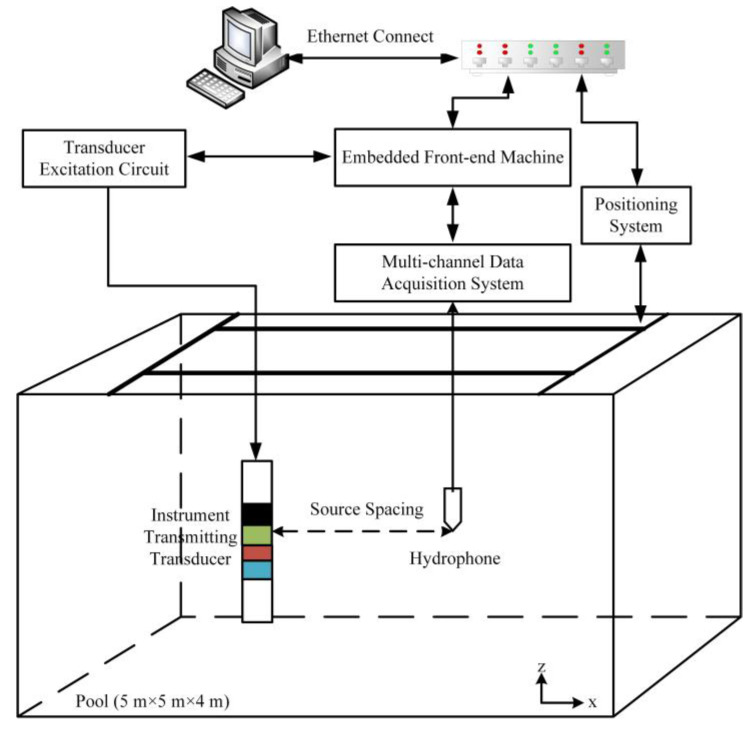
Schematic diagram of the transmitting transducer performance test.

Figure 10 shows the diagram of the performance test for the transmitting transducer. The measurement system is mainly composed of seismoelectric logging instrument, positioning control system, multi-channel data acquisition system, PC controller and BandK 8013 standard hydrophone. The synchronization signal of the multi-channel data acquisition system was provided by the main control electronic circuit of the instrument. The experiment was performed in a pool (5 m × 5 m × 4 m) filled with water. The excitation signal pulse width of the instrument transmitting transducer was 35 µs, and the excitation voltage was about 3000 V.

**Figure 11:**
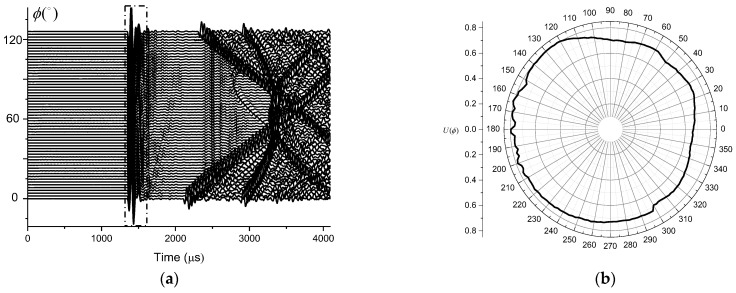
(**a**) The waveforms received by the hydrophone (0° to 120°). (**b**) Horizontal directivity diagram of the transmitter.

We first measured the horizontal directivity of the transducer. Due to experimental limitations, the circumferential horizontal directivity measurement could not be completed in one run. Because of that, the experiment was divided into four parts. In order to improve the accuracy of the experiment, the angle range in each part of the experiment was 120°, with the step angle being 2°. The overlapped data were used for verification and for combining datasets. During the experiment, the angle between the oil injection hole of the instrument and the hydrophone was 0°. The positioning system was controlled to enable a hydrophone scan of 120° around the measurement. The range of the first measurement was from 0° to 120°. After that measurement, the instrument was turned 30 degrees counterclockwise and the measurement was repeated. Subsequent measurements were performed using the same procedure with the fourth measurement being from 270° to 390°. The combined results of these measurements are shown in Figure 11a.

**Figure 12:**
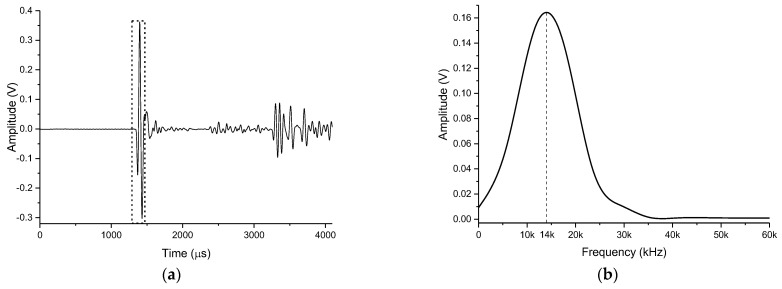
The (**a**) waveform registered at a zero angle and (**b**) its Fourier spectrum.

The overlapping part of the four measurements was reasonably selected, and the first arrival peak-to-peak value of the received waveforms at different angles was calculated to be within the range of 1300 µs to 1600 µs. The horizontal directivity of the transmitting transducer is shown in Figure 11b. According to the obtained results, the directionality of the transmitting transducer instrument had an approximately circular shape in the horizontal direction, which indicates that the transducer has no obvious directionality in the horizontal direction and can radiate acoustic energy uniformly to the borehole wall. Figure 12 shows the result of the Fourier transform of the waveform received at a zero angle defined since the time of its first arrival. According to Figure 12b the dominant frequency of this waveform was about 14 kHz.

**Figure 13:**
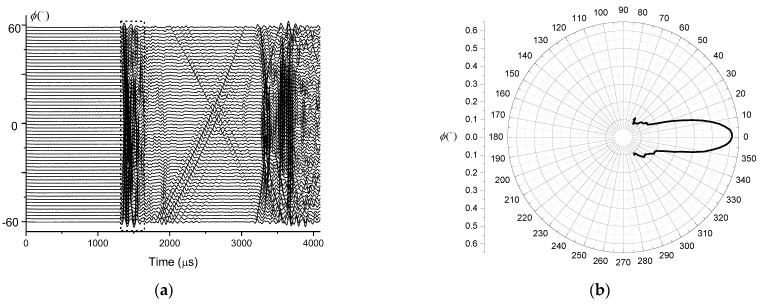
The (**a**) waveform without delay and (**b**) vertical directivity of the transmitter.

Since the transmitting transmitter has no obvious directivity in the horizontal direction, any position in the horizontal direction can be selected to test the vertical directivity. For this test we set the transmitting transducer to the normal operating mode and adjusted the distance to the source to 2 m. The hydrophone was moved in the vertical direction and the measurement was carried out in the range of 60° above and below with the step angle of 2°. A total of 61 groups of data were obtained during the experiment. The obtained waveforms are shown in Figure 13a. For each waveform, the vertical directivity of the transmitting transducer can be calculated by extracting the peak-to-peak value of the first arrival between 1300 µs and 1600 µs at the time. The result of these computations is shown in Figure 13b.

According to Figure 13, the major lobe direction of the transmitting transducer in the vertical plane is 0°, and there is no obvious side lobe. This indicates that the transmitting transducer has certain directivity in the vertical plane, and the radiated energy can be concentrated in the formation. Combining the horizontal and vertical directivity, the transmitting transducer of the seismoelectric logging detector can be considered an appropriate sound source.

### 3.2. Beam Deflection Experiment of Two-Element Linear Phased Array

The acoustic emission probe of the seismoelectric logging tool uses a two-element linear phased array, and each array element includes three monopole transducers. These three transducers share an excitation signal and work at the same time to enhance the radiation energy. When the linear phased array elements work simultaneously, the acoustic beam penetrates the formation in the horizontal direction. When the linear phased array elements work sequentially, the beam will be deflected, and the main lobe angle of the radiated acoustic beam will also be deflected to a certain extent. This can make the radiated energy more biased towards the receiving transducer, thereby enhancing the intensity of the acoustoelectric conversion signal and improving the signal-to-noise ratio of the received signal. The deflection angle is given by Equation (7) [36].

(7) $$\Delta \tau =d\;sin\theta_{s}/c$$

where Δτ is the radiation delay time of two array elements, *d* is the center distance between the two elements (200 mm), *c* is the wave velocity in water with a value of 1450 m/s and $\theta_{s}$ is the vertical directional main lobe deflection angle.

**Figure 14:**
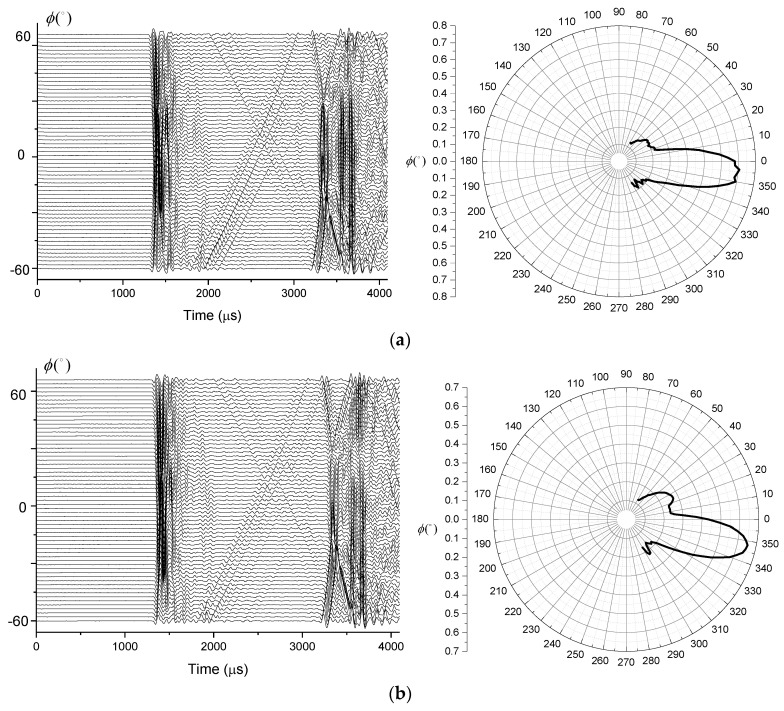

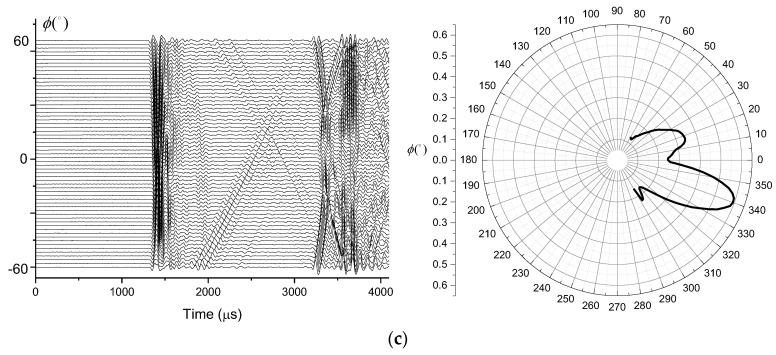
The waveform (**left**) and vertical directivity (**right**) for a (**a**) 10 µs (**b**) 20 µs (**c**) 30 µs delay.

During the experiment, the working delay times of the two groups of transducers were set to 10 µs, 20 µs and 30 µs, respectively. According to the used vertical directivity measurement protocol, 61 datasets were measured for each delay time, and the first arrival peak-to-peak value of each measurement was extracted to calculate the vertical directivity. The received waveforms and the vertical directivity for different delay times are shown in Figure 14.

**Figure 15:**
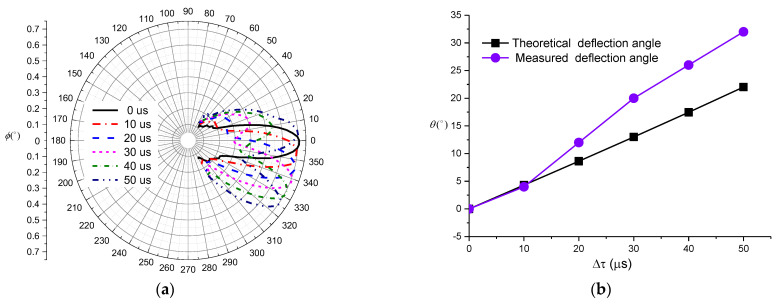
(**a**) Normalized vertical directivities of linear phased arrays. (**b**) Measured and theoretically computed deflection angles.

According to Figure 14, the deflection angle of the main lobe of the transmitting transducer beam was gradually increasing with the increase in radiation delay. When the radiation delay was greater than 20 µs, the side lobes of the radiated sound beam increased rapidly. At 50 µs of delay, the magnitude of the side lobe exceeded the magnitude of the main lobe. The comparison of the normalized vertical directivity of the transducer for different delays and the theoretically calculated deflection angle are shown in Figure 15. According to the obtained data, the actual main lobe deflection angle exceeds the theoretically computed angle when the radiation delay is greater than a certain value between 10 µs and 20 µs. Based on the comparison of the actual measured waveform and deflection angle, the radiation delay between the two array elements cannot be too large during actual logging, so that the delay should be kept below 20 µs.

### 3.3. Evaluating the Performance of the Receiving Transducer of the Seismoelectric Logging Detector

**Figure 16:**
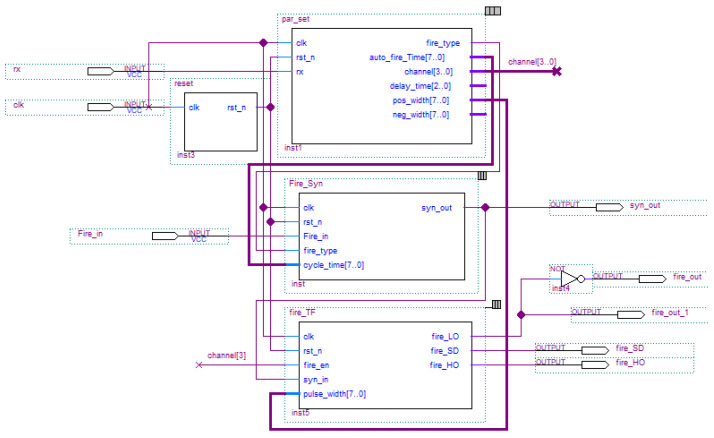
The CPLD module diagram of the transformer excitation control program.

**Figure 17:**
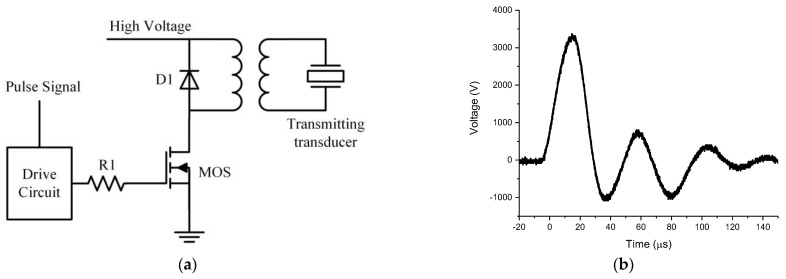
(**a**) Schematic diagram of transformer excitation circuit. (**b**) Driving waveform of the transformer excitation.

In the performance study of the seismoelectric logging detector, an external acoustic transducer is required to generate the excitation signal while the instrument detects the waveforms. Because of that, the excitation control circuit of a multi-channel transducer transformer was designed (see Figure 16). The excitation circuit uses a CPLD (complex programmable logic device) as the control module to generate the logic control and synchronization signals. The logic signal output by the CPLD is isolated by the isolation chip IS0122A, and the on/off of the MOS tube is controlled by the high-voltage driving circuit to generate an excitation signal to control the transmitting transducer. After the positive pulse logic signal with a specific width passes through the driving circuit, it controls the on/off state of the MOS tube to generate a high-voltage pulse. Then the high-voltage pulse is loaded onto the transducer through the pulse transformer to make the transducer work. The transformer’s excitation mode can output up to a 3400 V pulse signal. A schematic diagram of transformer excitation is shown in Figure 17. 

**Figure 18:**
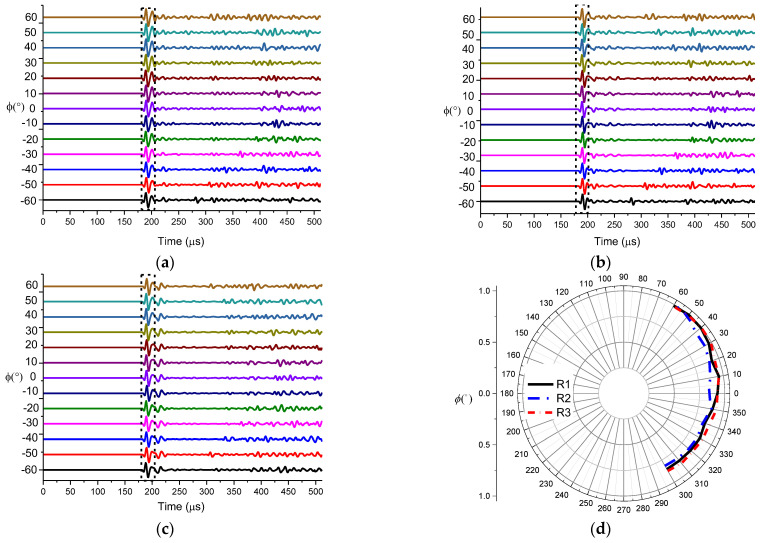
Waveforms received by transducer (**a**) R1, (**b**) R2, (**c**) and R3. (**d**) Normalized horizontal directivities of three transducers.

The seismoelectric logging detector has three receiving transducers. When measuring the characteristics of the receiving transducer, the multi-channel transducer excitation circuit was used to excite the external monopole transmitter transducer according to the transformer mode. The pulse width of the excitation signal was 30 µs and the excitation voltage is 3400 V. The acoustic signals were received by the instrument. The synchronous signal of the excitation circuit comes from the main control electronic circuit of the instrument. Through measurement, thirteen groups of waveforms were received on each transducer, and the peak-to-peak value of the first wave of each receiving waveform was calculated respectively to obtain the horizontal directivity of the receiving transducer. The results are shown in Figure 18.

**Figure 19:**
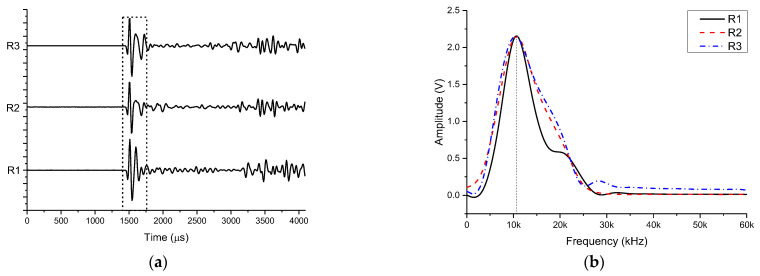
The (**a**) waveform and (**b**) spectrum curve of the receiver.

According to Figure 18d, the sensitivity of used three receiving transducers was uniform in the range of vertical angles from −60° to 60°. Since the measurement positions were randomly selected, it can be concluded that the receiving ability of the receiving transducer in the horizontal circumferential direction is also uniform. The signals of the three receiving transducers facing the transmitting transducer in the vertical direction (see Figure 19a), were taken and a Fourier transform was performed on the first wave. The result is shown in Figure 19b.

According to Figure 19a, the shapes of the direct waves received by each channel were similar, with some difference in the measured amplitudes. Using the curve length ratio method, the arrival time of the first wave was calculated to be 1444 µs (see Equation (8)).

(8) $$C=\frac{\sum_{t=T_{0}}^{T_{2}}\sqrt{{\left(x\left(t+\Delta t\right)-x\left(t\right)\right)}^{2}+\Delta t^{2}}}{\sum_{t=T_{1}}^{T_{0}}\sqrt{{\left(x\left(t+\Delta t\right)-x\left(t\right)\right)}^{2}+\Delta t^{2}}}$$

where △*t* is the sampling interval, *x(t)* is the collected waveform signal, *T*_1_ is the starting point of the window, *T*_0_ is the middle point of the window and *T*_2_ is the end point of the window. The calculated peak-to-peak value is the difference between the maximum peak and the minimum trough of the target wave packet.

The maximum direct peak-to-peak value of the three receiving transducers was 841 mV, the minimum was 822 mV, and the average was 832.3 mV. The shape of each spectrum curve was about the same, and the dominant frequency was about 10.4 kHz (see Figure 18b). Table 1 lists the peak-to-peak values of the direct waves received by the three receiving stations and their normalized values. According to the provided data, the normalized peak-to-peak values of the three receiving stations were all greater than 97%, and the consistency within the station was relatively high.

### 3.4. Field Test Data Analysis

The laboratory test results show that the receiving and transmitting performance of the seismoelectric logging tool meets the further test requirements. In order to verify the effectiveness of instrument data acquisition, we conducted actual measurements in a sand–shale well (light cement slurry, open hole, vertical well with a maximum depth of 3000 m) section at an oilfield in northern China, and processed the obtained data. All data were acquired under the acoustic excitation–acoustoelectric receiving mode. According to the seismoelectric logging data of well section 1740–1850 m in the XX well (XX represents the name of the measured well), the energy of accompanying conversion and acoustic Stoneley wave signals were calculated according to Equation (9) [37].

(9) $$\left\{ \begin{array}{l} E_{AA}=\sum_{t_{AA}}^{t_{AA}+L}W^{2}(t)\cdot \Delta t\\ E_{AE}=\sum_{t_{AE}}^{t_{AE}+L}D^{2}(t)\cdot \Delta t \end{array}\right.$$

where *E_AA_* is the energy of the acoustic Stoneley wave, *E_AE_* is the energy of seismoelectric signal accompanying conversion Stoneley wave, *W*(*t*) is magnitude of the received acoustic wave signal, *D*(*t*) is magnitude of the potential difference signal and Δ*t* is the sampling interval.

**Figure 20:**
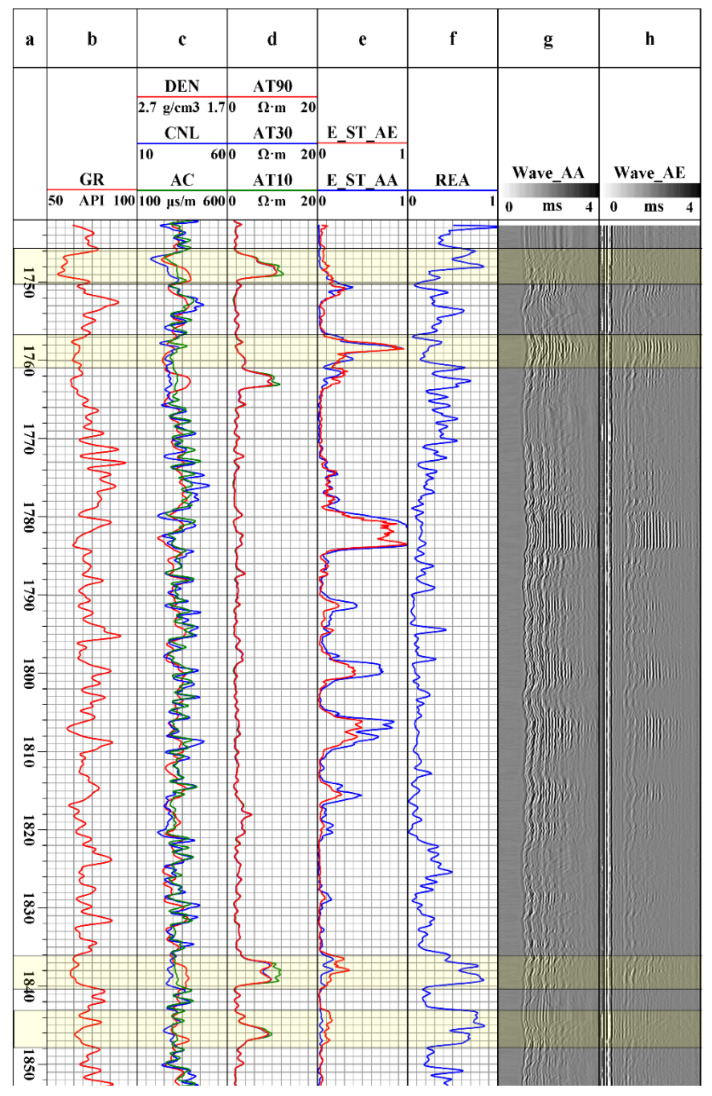
Data processing results of seismoelectric logging signals in some well sections.

In order to simplify the comparison, the energy curve was normalized to its maximum value. The calculation results are shown in Figure 20. In the figure, the first channel represents depth (a), the second channel is the natural gamma curve (b), the third channel contains density, neutron and sonic logging data (c), and the fourth channel is the array induced resistivity log (d). In the fifth channel, the accompanying conversion Stoneley wave signal energy (e, red curve) and Stoneley wave signal energy (e, blue curve) are included. We put the ratio of the accompanying Stoneley wave to the acoustic Stoneley wave on the sixth channel (f). The seventh channel shows the variable density diagram of the acoustic signal waveform (g), and the eighth channel shows the variable density diagram of the seismoelectric signal waveform (h).

According to the data on the fifth channel of Figure 20, the energy of the accompanying mode seismoelectric signals was consistent with the acoustic signals. For the well section with strong acoustic signal energy, the corresponding accompanying seismoelectric signal energy was also strong. This feature of the accompanying seismoelectric signals was obviously manifested in all the measured well sections. In order to reflect the unique information of acousto-electric signals better, we took the energy of the accompanying conversion and acoustic Stoneley wave signals as a ratio (REA *= E_AE_*/*E_AA_*). The REA is the electro-acoustic energy ratio of the accompanying conversion Stoneley wave. This ratio can represent the acoustic and electrical conversion energy of the formation to a certain extent, as shown in the sixth channel of Figure 20. Comparing the REA with conventional logging curves such as natural gamma, porosity and resistivity, it was found that there was a good correlation between them. When the well section has the characteristics of high REA, low natural gamma value, high porosity and a high resistivity curve while the amplitude difference between the deep and shallow resistivity curves is obvious, it may represent a reservoir interval with good porosity and permeability. This phenomenon can be explained from the generation mechanism of the seismoelectric effect. A formation with large porosity has good permeability. When the acoustic disturbance propagates to the formation, the more ions involved in relative migration in the rock, the higher the acoustic-electric conversion efficiency of the formation. The layers with obvious amplitude differenced in the resistivity curve in this well section show that the deep resistivity was less than the shallow resistivity, which was judged to be a water layer. There were definite *E_AA_* and *E_AE_* peaks at 1780, 1800, and 1805 m, which may be due to excessive changes in salinity (decreasing sharply). According to the numerical simulation study by Duan et al. [38], the amplitude of a seismoelectric interface-converted wave increases with the decrease of the fluid salinity. Therefore, the instrument was suitable for measurement in an environment with low salinity. As seismoelectric logging technology is under development, we are still studying the meaning of more details from the data obtained.

## 4. Discussion and Conclusions

In this paper, a new seismoelectric logging detector that can evaluate various geological parameters was designed. Aiming at the latest developed instrument, a transducer excitation circuit based on CPLD was designed, and a performance test was carried out in laboratory and field environments. It was proven by the experiments that the transmitting transducer instrument can radiate acoustic energy uniformly into the formation in the circumferential direction, and can transmit acoustic energy into the formation in the vertical direction in the form of a flat plate. The two groups of transmitting transducers form a binary phase-controlled linear array, and realize the deflection of the main lobe of the radiation beam. According to the performance test results of the receiving transducer, the instrument could receive the circumferential acoustic energy uniformly. The transducers of the receiving sound system had good consistency in the measured waveform amplitude and spectrum, and the comprehensive consistency of all channels was more than 97%. Through further analysis, the energy received by the phase-controlled receiving subarray was stronger than that received by a single array element. In conclusion, the seismoelectric logging detector has appropriate transmitting and receiving transducers.

During the following instrument improvement, it is necessary to further enhance the signal transmission power and improve the array structure of the transducer. Directional radiation and reception can be realized by controlling the amplitude and phase of the transducer radiation and the received acoustic signal. Phased array-receiving subarrays with different array elements have pointing characteristics. By setting the delay time, the dominant receiver can be accurately adjusted to improve the detection ability of weak seismoelectric signals.

## Figures and Tables

**Table 1:** Signal consistencies among stations R1 to R3.

Receiving Transducer	Peak-to-Peak Value (mV)	Normalized Value	Arrival Time of the First Wave (µs)	Dominant Frequency (kHz)
R1	834	0.992	1449	10.4
R2	822	0.977	1440	10.4
R3	841	1	1443	10.3

## Data Availability

The study did not report any data.
